# Pharmacokinetics of the soluble guanylate cyclase stimulator riociguat in individuals with renal impairment

**DOI:** 10.1186/2050-6511-14-S1-P22

**Published:** 2013-08-29

**Authors:** Reiner Frey, Corina Becker, Sigrun Unger, Anja Schmidt, Georg Wensing, Wolfgang Mueck

**Affiliations:** 1 Clinical Pharmacology, Bayer Pharma AG, Pharma Research Centre, Wuppertal, Germany; 2 Global Biostatistics, Bayer Pharma AG, Pharma Research Centre, Wuppertal, Germany

## Background

Riociguat is the first oral, soluble guanylate cyclase stimulator under review for the treatment of pulmonary hypertension (PH), a progressive, ultimately fatal disease [1-7]. This pooled analysis of two studies evaluated the pharmacokinetics of riociguat and its metabolite M1 (BAY 60-4552) in individuals with and without renal impairment. The safety and tolerability of riociguat were also assessed.

## Methods

Two non-randomized, non-blinded, observational studies with group stratification were conducted in a single centre in Germany, following Good Clinical Practice and relevant industry guidelines [8,9]. Participants were assigned to one of four renal function groups according to their creatinine clearance (CL_CR_): group 1, CL_CR_ > 80 mL/min; group 2, CL_CR_ 50–80 mL/min; group 3, CL_CR_ 30–49 mL/min; group 4, CL_CR_ < 30 mL/min. In the first study, individuals in group 4 received riociguat 0.5 mg; all other participants in both studies received riociguat 1 mg (single tablet doses). Pharmacokinetic parameters were assessed using dense sampling.

## Results

**Figure 1:**
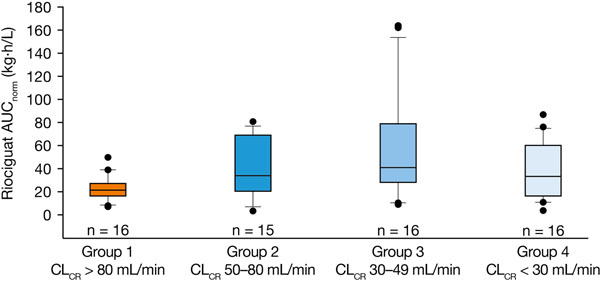
Box-and-whisker plot of riociguat AUC_norm_ (kg·h/L) after a single oral dose of riociguat. In the first study, individuals with severe renal impairment (group 4) received riociguat 0.5 mg; all other participants in both studies received riociguat 1 mg . Box, 25–75th percentile; vertical line, 10th–90th percentile; horizontal line, median; more extreme values are plotted as points; individuals eligible for pharmacokinetic analysis, n = 63; AUC_norm_, area under the plasma concentration–time curve from time 0 to infinity divided by dose per kilogram of body weight for total riociguat.

Sixty-three participants (40 men and 23 women; mean age, 61.3 years [range, 36–78 years]) completed the study and were eligible for pharmacokinetic analysis. Riociguat was rapidly absorbed; median time to reach maximum concentration in plasma (t_max_) (C_max_) was 1 hour in all four groups. Mean half-life of total riociguat was longer in groups 2–4 (9.5–11.4 hours) than in group 1 (6.2 hours) (Table 1), and renal clearance of riociguat decreased with decreasing renal function. Mean exposure to total riociguat (area under the concentration–time curve divided by dose per kilogram of body weight [AUC_norm_]) was 42.7–104.3% higher in groups 2–4 than in group 1 (Table 1, Figure 1). However, exposure was highly variable in groups 2–4 and the exposure ranges in all groups overlapped (Figure 1). Exposure to riociguat did not increase strictly in parallel with decreasing CL_CR_. Results for unbound riociguat and M1 were similar to those for total riociguat and M1. No serious or severe adverse events were reported. Headache was the most common drug-related adverse event. No changes in safety or tolerability were detected with decreasing CL_CR_. Riociguat C_max_ and AUC ranges in patients with renal impairment overlapped those previously observed in healthy volunteers and patients with PH [2,3].

**Table 1:** Pharmacokinetic parameters of riociguat in healthy participants and in individuals with mild, moderate or severe renal impairment

Parameter	Group 1 (CR_CL_ > 80 mL/min) n = 16	Group 2 (CR_CL_ 50–80 mL/min) n = 15	Group 3 (CR_CL_ 30–49 mL/min) n = 16	Group 4^a^ (CR_CL_ < 30 mL/min) n = 16
AUC, μg·h/L	245.7 (51)	347.5 (111)	499.0 (110)	523.0 (70.4)^b^
C_max,_ μg/L	36.6 (17)	44.2 (21)	42.0 (32)	40.56 (37.8)^b^
AUC_norm_, kg·h/L	20.6 (56)	29.4 (126)	42.1 (109)	29.7 (102)
C_max,norm_, kg/L	3.07 (17)	3.48 (25)	3.54 (30)	2.97 (40)
t_½_, h	6.19 (50)	10.1 (116)	11.4 (103)	9.52 (75)

^a^In the first study, individuals with severe renal impairment (group 4) received riociguat 0.5 mg; all other participants in both studies received riociguat 1 mg.

^b^AUC and C_max_ values shown for individuals with severe renal impairment (group 4) are taken from the second study (n = 8), in which individuals with severe renal impairment received riociguat 1.0 mg.

Values are geometric means (percentage coefficient of variation). AUC, area under the plasma concentration–time curve from time 0 to infinity; AUC_norm_, AUC divided by dose per kilogram of body weight for total riociguat; C_max_, maximum concentration in plasma; C_max,norm_, C_max_ divided by dose per kilogram of body weight for total riociguat; t_½_, terminal elimination half-life for total riociguat.

## Conclusion

Exposure to riociguat was higher in individuals with renal impairment (CL_CR_ 15–80 mL/min) than in controls; particular care should be exercised during individual dose titration in patients with renal impairment.

## References

[B1] Stasch JP Pacher P Evgenov OV Soluble guanylate cyclase as an emerging therapeutic target in cardiopulmonary disease Circulation 2011 123 2263 2273 10.1161/CIRCULATIONAHA.110.981738 21606405 PMC3103045

[B2] Frey R Mück W Unger S Artmeier-Brandt U Weimann G Wensing G Single-dose pharmacokinetics, tolerability and safety of the soluble guanylate cyclase stimulator BAY 63-2521; an ascending-dose study in healthy male volunteers J Clin Pharmacol 2008 48 926 934 10.1177/0091270008319793 18519919

[B3] Grimminger F Weimann G Frey R Voswinckel R Thamm M Bölkow D Weissmann N Mück W Unger S Wensing G First acute haemodynamic study of soluble guanylate cyclase stimulator riociguat in pulmonary hypertension Eur Respir J 2009 33 785 792 10.1183/09031936.00039808 19129292

[B4] Ghofrani HA Hoeper MM Halank M Meyer FJ Staehler G Behr J Ewert R Weimann G Grimminger F Riociguat for chronic thromboembolic pulmonary hypertension and pulmonary arterial hypertension: a phase II study Eur Respir J 2010 36 792 799 10.1183/09031936.00182909 20530034

[B5] Ghofrani H Galie N Grimminger F Humbert M Keogh A Langleben D Kilama MO Neuser D Rubin L Riociguat for the treatment of pulmonary arterial hypertension: a randomized, double-blind, placebo-controlled study (PATENT-1) Chest 2012 142 1027A 10.1378/chest.146279923032451

[B6] Ghofrani H Grimminger F Hoeper M Kim N Mayer E Neuser D Pena J Simonneau G Wilkins M Riociguat for the treatment of inoperable chronic thromboembolic pulmonary hypertension: a randomized, double-blind, placebo-controlled study (CHEST-1) Chest 2012 142 1023A 10.1378/chest.1462924

[B7] Hurdman J Condliffe R Elliot CA Davies C Hill C Wild JM Capener D Sephton P Hamilton N Armstrong IJ Billings C Lawrie A Sabroe I Akil M O'Toole L Kiely DG Aspire Registry: assessing the spectrum of pulmonary hypertension identified at a referral centre Eur Respir J 2012 39 945 955 10.1183/09031936.00078411 21885399

[B8] Guidance for Industry. Pharmacokinetics in Patients with Impaired Renal Function - Study Design, Data Analysis, and Impact on Dosing and Labeling [http://www.fda.gov/downloads/Drugs/GuidanceComplianceRegulatoryInformation/Guidances/ucm072127.pdf]

[B9] Note for Guidance on the Evaluation of the Pharmacokinetics of Medicinal Products in Patients with Impaired Renal Function [http://www.ema.europa.eu/docs/en_GB/document_library/Scientific_guideline/2009/09/WC500003123.pdf]

